# Assessment of Bone Mineral Density in Children with Developmental Dysplasia of the Hip Joint: Possible Risk Factors for Osteopenia and Osteoporosis

**DOI:** 10.3390/medicina61101727

**Published:** 2025-09-23

**Authors:** Maha A. Al Slateen, Alaa Ibrahim, Turki Abualait, Ammar Alomran, Sarah Alshahwan, Mariam Alsomali, Mohammed S. Abdelsalam

**Affiliations:** 1 Department of Physical Therapy, Khamis Mushait Maternity and Children Hospital, Ministry of Health, Khamis Mushait 62454, Saudi Arabia; 2 Department of Physical Therapy, College of Applied Medical Sciences, Imam Abdulrahman Bin Faisal University, Dammam 34212, Saudi Arabia; aiibrahim@iau.edu.sa (A.I.); tsabualait@iau.edu.sa (T.A.); 3 Department of Orthopedic, College of Medicine, Imam Abdulrahman Bin Faisal University, Dammam 31441, Saudi Arabia; aomran@iau.edu.sa; 4 Department of Diagnostic Radiology, College of Medicine, Imam Abdulrahman Bin Faisal University, Dammam 31441, Saudi Arabia; sshahwan@iau.edu.sa; 5 Department of Radiology, King Fahad University Hospital, Khobar 34445, Saudi Arabia; mssomali@iau.edu.sa; 6 Department of Physical Therapy for Musculoskeletal Disorders and Its Surgery, Faculty of Physical Therapy, Cairo University, Cairo 11432, Egypt; m.shawki@pt.cu.edu.eg

**Keywords:** developmental dysplasia of the hip, bone mineral density, osteopenia, osteoporosis, motor performance, weight bearing, physical activity, muscle strength

## Abstract

*Background and Objectives:* Developmental dysplasia of the hip (DDH) in children can result in long-term skeletal complications, including a reduced bone mineral density (BMD) and an increased risk of osteoporosis. This study aimed to evaluate BMD in children with DDH and to identify clinical, functional, and nutritional factors associated with reduced BMD. *Materials and Methods:* A cross-sectional study was conducted on 25 children aged 5–10 years with confirmed DDH. Bone mineral density was measured at the total body, subtotal, and lumbar spine using dual-energy X-ray absorptiometry (DXA), and Z-scores were calculated. Functional assessments included isometric muscle strength, weight-bearing symmetry, and physical activity measured via accelerometry. Demographic data and daily calcium intake were recorded. Correlation and multiple linear regression analyses were performed to determine predictors of BMD. *Results:* Most participants exhibited normal growth and mobility, with mild asymmetries in limb strength and length. The mean total BMD was within normative ranges, whereas the lumbar spine Z-score (−1.41 ± 1.72) was mildly reduced. BMD positively correlated with age, anthropometric measures, weight-bearing capacity, and calcium intake, and negatively correlated with a family history of osteoporosis. Multiple regression analysis identified the muscle strength symmetry index as the strongest independent predictor of BMD across all sites (subtotal Z-score: β = 1.000, *p* < 0.001; total Z-score: β = 0.425, *p* = 0.023; lumbar Z-score: β = 0.499, *p* = 0.014). Physical activity levels showed no significant associations with BMD. *Conclusions:* Children with DDH generally demonstrate preserved overall BMD; however, mild lumbar spine deficits may occur. Muscle strength symmetry appears to be the most influential modifiable factor for optimizing bone health in this population, highlighting the importance of targeted physiotherapy interventions.

## 1. Introduction

Developmental dysplasia of the hip (DDH) is a prevalent musculoskeletal disorder in infants, characterized by abnormal development of the acetabulum and/or femoral head, leading to improper joint alignment [1]. Multiple risk factors have been identified, including breech presentation, female sex, firstborn status, positive family history, specific racial backgrounds, oligohydramnios, intrauterine malposition, breech delivery, environmental influences, and elevated maternal estrogen levels [2]. If not promptly diagnosed and managed, DDH can result in serious long-term consequences, such as osteoarthritis, avascular necrosis, and impaired motor function [3]. Research has shown that individuals with a history of DDH may experience physical limitations, reduced participation in sports, and diminished quality of life [4]. Furthermore, unilateral DDH treatment has been associated with altered balance control strategies, including shifts in the center of mass and center of pressure during walking, which affect frontal plane stability during standing and sagittal plane control during the swing phase of gait [5]. Emerging evidence also indicates that children with DDH may exhibit reduced bone mineral density (BMD), potentially predisposing them to osteopenia and osteoporosis later in life [6], thereby increasing their lifetime risk of hip fractures [7].

Bone health during childhood is a critical determinant of lifelong skeletal integrity, as the majority of peak bone mass is achieved during childhood and adolescence [8]. This period represents a unique window of opportunity in which optimal bone accrual can significantly reduce the risk of osteoporosis and fractures later in life. The process of bone mineral accumulation is regulated by a complex interplay of factors, including physical activity, nutrition, hormonal status, and genetic predisposition [9]. Weight-bearing activities, such as walking, running, and jumping, are particularly important, as they stimulate osteogenesis through mechanical loading, thereby enhancing bone density and reducing bone resorption. Adequate dietary intake of calcium and vitamin D further supports bone mineralization, while genetic factors influence individual variability in accrual rates and ultimate peak bone mass.

In the context of DDH, assessing bone mineral density is essential to understanding the potential consequences of altered hip biomechanics on skeletal development. Although structural abnormalities in DDH have been extensively described, the relationship between DDH and long-term bone health, particularly the risk of osteopenia and osteoporosis, remains insufficiently explored. Only a limited number of studies have examined BMD in children with DDH, and even fewer have evaluated its association with functional parameters of motor performance, such as weight-bearing symmetry, physical activity levels, and muscle strength [10].

Elucidating these associations is crucial for early identification of children at risk for compromised bone health and for implementing timely interventions to prevent future complications. The present study aims to assess BMD in children diagnosed with DDH, identify potential risk factors for reduced bone density, and investigate the relationship between BMD and key motor performance indicators. By integrating biomechanical and functional assessments, this research seeks to contribute to a more comprehensive understanding of musculoskeletal health in DDH, ultimately guiding clinical strategies to optimize bone development and reduce lifelong fracture risk.

## 2. Materials and Methods

A cross-sectional study was conducted from January to June 2025 in the Orthopedic Department at King Fahd Hospital of the University (KFHU). The study included children of both genders, aged 3–10 years, with a confirmed DDH diagnosis, who were undergoing or had completed treatment. All participants were prepubertal; therefore, pubertal hormonal status was not assessed. Children with comorbidities that may affect bone density (e.g., vitamin D deficiency, hyperparathyroidism) or mobility (e.g., cerebral palsy, muscular dystrophy) were excluded. No participant had a history of systemic, local, or inhaled corticosteroid use, as verified through medical records and parental interviews. Additionally, children undergoing treatments that could influence bone density or mobility were excluded.

The sample size was determined using G*Power 3.1 software, based on effect sizes reported in previous studies assessing bone mineral density differences in pediatric orthopedic conditions [11,12]. Using an anticipated effect size of f = 0.50 (large effect), an alpha level of 0.05, and a statistical power of 0.95 for a two-tailed test, the minimum required sample size was calculated to be 22 participants. To account for potential dropouts or incomplete data, an additional 20% was added, resulting in a target sample size of 26 participants.

Each participant’s data were documented on a personalized and confidential data sheet. Prior to examination, participants underwent an evaluation to assess demographic and anthropometric parameters, including age, gender, birth order, twinning, family history of DDH, mode of delivery (cesarean or vaginal), birth presentation, maturity, body mass index (BMI), and the presence of associated anomalies such as foot deformities, scoliosis, developmental delay, speech issues, or vision problems. Clinical characteristics were also recorded, including type of treatment intervention (conservative or surgical), limb length discrepancy (inter-side difference between thighs and legs), and circumferential measurement discrepancies.

Participants underwent dual-energy X-ray absorptiometry (DXA) scanning (HOLOGIC Discovery-Ci DXA scanner (Hologic, Inc./United States (Bedford, MA, USA) to measure areal bone mineral density (BMD), representing bone mass per unit area. BMD was expressed in grams of hydroxyapatite per square centimeter (g/cm^2^). DXA is an accurate and widely used method for assessing bone mineral density [13]. In this study, BMD Z-scores were calculated using chronological (decimal) age- and sex-specific reference standards provided by the HOLOGIC software. Version 5.6.1.3 rev 006 Height adjustment was not applied, as participants exhibited normal growth within WHO reference standards. Total and subtotal Z-scores (whole-body-less-head) as well as lumbar spine Z-scores were assessed. The total Z-score included arms, legs, trunk, and head, whereas the subtotal Z-score included all body regions except the head, consistent with manufacturer protocols.

Physical activity (PA) levels were assessed using the ActiGraph accelerometer (Bluetooth^®®^ Smart wGT3X-BT, Kirkland, WA, USA). Data were transferred via USB to the ActiLife software Version 6.13.6 for processing and analysis. The ActiGraph is a valid and reliable instrument for evaluating physical activity [14]. Activity intensity was categorized as sedentary (<800 counts per minute [cpm]), light (800–3200 cpm), moderate (3200–8200 cpm), or vigorous (≥8200 cpm) [15]. Total steps and step rate (steps per minute) were also recorded.

Weight-bearing in a static position was measured using digital scales [16]. Weight-bearing symmetry was assessed by comparing the weight supported on each side, and the absolute symmetry index (SI) was calculated; an SI value of 1 indicates perfect symmetry.

Isometric muscle strength (IMS) of all hip and knee muscles bilaterally was measured using a handheld dynamometer (Commander Power-Track II, JTECH Medical, Midvale, UT, USA) following validated procedures and normative references [17]. Muscle groups measured included hip flexors, extensors, abductors, adductors, and knee flexors and extensors. Indices calculated included:

- *Non-affected strength index: sum of all non-affected muscle group scores*
- *Affected strength index: sum of all affected muscle group scores*
- *Muscle strength symmetry index: ratio of the sum of affected muscle group scores to non-affected muscle group scores*

Calcium intake was assessed via a parental survey using the International Osteoporosis Foundation (IOF) Calcium Calculator [18]. Guidance on achieving recommended calcium intake was provided if the estimated intake was below recommended levels.

All analyses were performed using SPSS software (version 27.0; SPSS Inc., Chicago, IL, USA). Continuous variables were checked for normality using the Shapiro–Wilk test, histogram inspection, and Q-Q plots. Pearson and Spearman correlation coefficients were used to assess relationships among BMD (total, subtotal, and lumbar spine Z-scores), anthropometric and clinical characteristics, and study outcomes. Multiple stepwise linear regression models were used to identify significant predictors of Z-scores. Statistical significance was set at *p* < 0.05.

## 3. Results

### 3.1. Demographic and Clinical Characteristics of Participating Children with DDH

Table 1 presents the demographic and clinical characteristics of participating children with DDH. The mean age of the sample was 8.48 ± 1.48 years. The sample comprised 25 children, with 76% being girls and 24% boys. The mean weight was 27.90 ± 9.96 kg, the mean height was 129.23 ± 8.94 cm, resulting in a mean body mass index (BMI) of 16.29 ± 4.10 kg/m^2^.

In terms of ambulation, the majority (96%) were able to walk independently, while only 4% required the use of a mobility device. Regarding birth order, 24% were firstborns, and 76% were not. Regarding the type of delivery, 44% were delivered via cesarean section and 56% via normal vaginal delivery. Breech presentation was observed in 16% of the children, while 84% had a normal presentation at birth.

Laterality showed that 96% of the cases were unilateral, while 4% were bilateral. The side affected was the right (60%) and left (36%), with 4% having bilateral involvement. A positive family history of DDH was present in 52% of cases.

Foot deformities were noted in 12% of the children, while 88% had no such abnormalities. Associated problems like spina bifida, arthrogryposis multiplex congenita, scoliosis, torticollis, speech problems, visual problems, etc., were reported in 16% of the sample. Treatment intervention included surgical procedures in 72% of cases, while 28% underwent conservative treatment.

The mean estimated daily calcium intake was 1083.20 ± 285.00 mg. On average, children achieved 93.37% ± 28.43% of the recommended intake.

### 3.2. Anthropometric Measurements, Physical Activity, and Isometric Muscle Strength

Table 2 represents the anthropometric measurements, physical activity, and Isometric muscle strength in the DDH children studied. The mean discrepancies between the non-affected and affected femoral and tibial lengths were 0.36 ± 0.47 cm and 0.34 ± 0.47 cm, respectively. Similarly, round measurements of the thigh and leg showed mean discrepancies of 0.36 ± 0.45 cm and 0.24 ± 0.41 cm, respectively.

The mean weight-bearing capacity was slightly lower on the affected side (13.38 ± 5.31 kg) compared to the non-affected side (14.26 ± 4.86 kg), with an interside difference of −0.42 ± 2.10 kg. The symmetry index had a mean of 0.97 ± 0.15.

Regarding physical activity parameters, the mean total step count was 35869.8800 ± 18235.558 steps with a step rate of 8.75 ± 3.951 steps per minute. In terms of activity classification, the average total sedentary time constituted 52.48% ± 10.56 of the total wear time. Light activities accounted for 42.67% ± 8.80, moderate activities for 4.61% ± 3.22, and vigorous activities for 0.14% ± 0.19 of the total activity time.

Regarding isometric muscle strength, the hip flexors had a mean strength of 18.70 ± 3.37 pounds on the non-affected side and 15.45 ± 3.23 pounds on the affected side. Hip extensors showed non-affected and affected side means of 20.30 ± 4.35 and 17.71 ± 3.80 pounds, respectively. Hip abductor strength averaged 17.64 ± 3.31 pounds on the non-affected side and 15.60 ± 2.89 pounds on the affected side, while hip adductors were measured at 19.63 ± 4.38 pounds on the non-affected side and 16.75 ± 3.25 pounds on the affected side.

Knee flexor strength was 15.40 ± 2.82 pounds for the non-affected side and 13.91 ± 2.88 pounds for the affected side. Knee extensors exhibited the highest individual muscle group values, with 20.75 ± 3.34 pounds (non-affected) and 18.52 ± 3.16 pounds (affected).

Additional strength indices included the non-affected strength index (112.42 ± 16.74 pounds), affected strength index (97.96 ± 15.58 pounds), and muscle strength symmetry index (0.87 ± 0.04 pounds).

### 3.3. Dual-Energy X-Ray Absorptiometry (DXA) Measurements

Table 3 presents dual-energy X-ray absorptiometry (DXA) measurements in participating children with DDH. The mean subtotal bone mineral content (BMC) was 611.59 ± 157.06 g, with a corresponding subtotal BMD of 0.6082 ± 0.0904 g/cm^2^. Head region measurements showed a mean BMC of 473.26 ± 123.24 g and a mean BMD of 1.7632 ± 0.1757 g/cm^2^. Total body values were also reported, with a mean total BMC of 993.65 ± 258.79 g and a total BMD of 0.8303 ± 0.0799 g/cm^2^.

Z-scores, which reflect deviation from age-matched normative values, are provided for subtotal, total, and lumbar (L1–L4) regions. The mean subtotal Z-score was −1.18 ± 1.58, while the total Z-score was 0.99 ± 1.16. In the lumbar spine (L1–L4), the mean BMC was 16.57 ± 5.04 g, and the mean BMD was 0.50 ± 0.11 g/cm^2^, with a lumbar Z-score of −1.41 ± 1.72.

### 3.4. Correlation Between Dual-Energy X-Ray Absorptiometry (DXA) Measurements and Independent Variables

Table 4 represents the correlation between Dual-energy X-ray absorptiometry (DXA) measurements and independent variables of the studied DDH children. Age had a positive correlation with the subtotal Z-score, total Z-score, and total lumbar Z-score, indicating that higher ages are associated with higher Z-scores (*p* < 0.05).

Anthropometric parameters such as weight, height, and BMI demonstrated significant positive correlations with the DXA subtotal and lumbar Z-scores (*p* < 0.05). Family history was positively correlated with the subtotal Z-score, total Z-score, and total lumbar Z-score (*p* < 0.05), indicating that positive family history is associated with lower Z-scores and bone mineral density.

Weight bearing on both affected and non-affected sides showed significant positive associations with the subtotal Z-score (*p* < 0.05). Additionally, weight-bearing on the affected side was positively correlated with the lumbar Z-score (*p* < 0.05).

Concerning calcium intake, the estimated daily calcium was positively correlated with subtotal Z-score, total Z-score, and total lumbar Z-score (*p* < 0.05). The percentage of estimated calcium was only positively correlated with the total Z-score. This indicates that calcium intake positively affects the bone mineral density and Z-scores in those children.

On the other hand, none of the physical activity characteristics were correlated with any of the DXA Z-scores (*p* > 0.05).

Regarding isometric muscle strength, among the measured muscle groups, only the affected knee flexors were positively correlated with the subtotal Z-score (*p* < 0.05). Neither the affected strength index nor the non-affected strength index recorded a significant association with any of the DXA Z-scores (*p* > 0.05). Interestingly, the affected/non-affected strength symmetry index was positively correlated with all measured Z-scores (*p* < 0.05), indicating that the increased strength asymmetry is associated with low bone mineral density.

### 3.5. Prediction of Dual-Energy X-Ray Absorptiometry (DXA) Z-Scores

Table 5 presents the results of multiple linear regression analyses conducted to identify predictors of Dual-energy X-ray absorptiometry (DXA) Z-scores in participating children with DDH. Separate models were constructed for the subtotal Z-score, total Z-score, and total lumbar (L1–L4) Z-score.

In the model predicting the subtotal Z-score, age (β = 0.117), BMI (β = 0.121), weight bearing on the affected side (β = 0.04), weight bearing on the nonaffected side (β = −0.207), estimated daily calcium intake (β = 0.001), family history (β = −0.058), affected knee flexors (β = −0.090), and strength symmetry index (β = 1.00) were included as predictors. The model was statistically significant (R^2^ = 0.95, *p* < 0.001), with only the strength symmetry index reaching individual significance.

For the total Z-score, age (β = −0.059), estimated daily calcium intake (β = 0.293), percentage of estimated daily calcium intake (β = 0.254), family history (β = 0.160), and strength symmetry index (β = 0.425) were examined. The model was statistically significant overall (R^2^ = 0.737, *p* < 0.001), while the strength symmetry index only reached individual significance.

The total lumbar Z-score model included age (β = 0.126), BMI (β = 0.019), weight bearing on the affected side (β = −0.002), estimated daily calcium intake (β = 0.156), family history (β = 0.280), and strength symmetry index (β = 0.499). This model was statistically significant (R^2^ = 0.762, *p* < 0.001), with the strength symmetry index emerging again as a significant predictor.

## 4. Discussion

This research provides an in-depth examination of the anthropometric, clinical, functional, and bone health attributes of children diagnosed with developmental dysplasia of the hip (DDH). The key findings drawn from our study are as follows: the overall bone mineral density (BMD) remained within standard clinical limits, lumbar spine Z-scores exhibited slight decreases, the strength of the affected limb was consistently inferior to that of the non-affected limb, and muscle strength symmetry was identified as the most crucial predictor of BMD.

This study offers a comprehensive analysis of the anthropometric, clinical, functional, and bone health characteristics of children with developmental dysplasia of the hip (DDH). The sex distribution in the sample (76% female) aligns with the well-documented female predominance in DDH, likely due to ligamentous laxity mediated by maternal hormones [19]. Though firstborn status and breech presentation are classic DDH risk factors [20], only 24% were firstborn in this cohort, while 16% had breech presentation, still within the expected range. Notably, 52% reported a positive family history, supporting the role of genetic predisposition in DDH [21].

Delivery mode was evenly distributed, with 44% delivered via cesarean section and 56% by vaginal delivery. Breech presentation was identified in 16% of participants, consistent with the strong association between breech delivery and DDH documented in previous studies [22]. All participants were born full-term, with no reported prematurity, consistent with the observation that DDH is more commonly associated with full-term infants [20].

Children in this study demonstrated normal somatic growth, with average weight, height, and BMI values falling within WHO reference ranges [23]. This supports prior evidence that DDH, when appropriately treated, does not hinder overall physical development [24]. However, mild asymmetries were noted between affected and unaffected limbs in femoral/tibial lengths and circumference measurements, likely reflecting residual structural differences post-treatment [25].

Weight-bearing distribution was nearly symmetrical (symmetry index = 0.97), though the affected limb bore slightly less weight, a subtle functional impairment also noted in previous research [26]. Functionally, most children were ambulatory (96%), reflecting early and effective intervention. A small subset had comorbidities such as clubfoot, scoliosis, or spina bifida, which are known to co-occur with DDH [27]. Nutritionally, calcium intake was close to the recommended level (93.4% of the Recommended Daily Calcium Intake), which is generally acceptable for bone health [28].

Using accelerometry, the study found that children with DDH engaged in moderate daily activity, yet moderate-to-vigorous physical activity (MVPA) was significantly below recommended levels [29]. Only 4.6% of wear time was spent in MVPA, and vigorous activity accounted for just 0.1%, potentially due to discomfort, overprotection, or altered biomechanics [29]. Low MVPA is concerning, as it may impair cardiovascular, skeletal, and psychosocial development [30]. These findings emphasize the importance of tailored physical therapy programs for children with DDH. The total steps and step rate in our sample averaged approximately 35,869 steps with a step rate of 8.75 steps per minute, providing an additional quantitative index of movement. Although direct comparison to normative data is limited by methodological differences, healthy school-aged children have been reported to achieve between 11,000 and 14,000 steps per day [31]. The comparatively lower step rates observed in our cohort may reflect subtle functional limitations, even in the presence of preserved ambulation.

DXA scans revealed that while total body BMD and BMC were within acceptable ranges, lumbar spine Z-scores were mildly reduced, indicating regional bone deficits [32]. Compared to previous studies in pediatric orthopedic populations like cerebral palsy [33] and scoliosis [34], our cohort showed slightly higher overall BMD and milder lumbar spine deficits. Differences may be due to methodological variations (DXA protocols, reference databases), sample characteristics (age, DDH severity, treatment stage), and context-specific factors such as nutrition, early diagnosis, and timely intervention, which may have mitigated bone health deterioration observed in other populations [35,36,37]. In general, findings are consistent with previous literature showing that children with orthopedic conditions, particularly those affecting weight-bearing joints such as DDH, may exhibit mild regional reductions in bone mass, especially in the lumbar spine and lower limbs [35]. Possible contributing factors include reduced mechanical loading of the affected hip due to gait asymmetry, pain avoidance, or residual functional limitations following surgery [36]. Mechanical strain is a well-known stimulus for bone modeling and accrual during childhood, as described by Frost’s mechanostat theory [37]. Therefore, children with DDH who exhibit altered loading patterns may have lower site-specific BMD. Importantly, no child had a Z-score below −2, the threshold for low bone mass for age [38], indicating overall satisfactory bone health, but with localized concerns requiring monitoring.

Assessment of isometric muscle strength revealed consistent weakness in the affected limbs, especially in the hip abductors, critical for gait stability [39]. The knee muscle groups similarly showed lower strength on the affected side. Although DDH is primarily a hip pathology, altered loading and compensatory movement patterns can influence distal muscle function as well [40]. The mean strength symmetry index indicated clinically significant asymmetry [41], though relatively mild. Compensatory movement patterns and altered neuromuscular control likely contribute to these deficits [42], underscoring the role of rehabilitation to restore bilateral strength balance.

This study demonstrates that bone mineral density (BMD) in children with DDH is determined by a combination of growth-related, biomechanical, nutritional, and genetic factors. Age showed a positive correlation with subtotal, total, and lumbar DXA Z-scores, reflecting the normal trajectory of bone accrual during growth, skeletal maturation, and pubertal changes [35,43]. Anthropometric measures such as weight, height, and BMI were also positively associated with BMD, consistent with evidence that larger body size enhances mechanical loading, which in turn stimulates bone modeling through mechanotransduction, in accordance with Wolff’s law and the mechanostat theory [37,44]. Functional mechanical loading, as reflected by the weight-bearing capacity on both limbs, was significantly correlated with higher Z-scores. This aligns with the concept that weight-bearing activities stimulate osteoblastic activity and promote bone strength [36]. In DDH, asymmetrical or reduced loading on the affected limb may compromise local bone development, while symmetrical loading appears protective, a finding consistent with other pediatric orthopedic populations [35].

Nutritional status, particularly total calcium intake, was strongly associated with subtotal, total, and lumbar Z-scores. Adequate calcium is crucial for hydroxyapatite formation and bone mineralization [45], and low intake during childhood has been linked to a reduced peak bone mass and increased fracture risk [35,46]. The stronger correlation with absolute calcium intake compared to the percentage of recommended intake suggests that total consumption remains a key determinant of bone health in this group. Even though children with a deficiency in vitamin D were not included, the status of vitamin D is still a vital factor in bone metabolism and could influence calcium intake. Consequently, future research should include both dietary calcium and vitamin D levels to offer a more comprehensive understanding of bone health in this group [47,48,49].

Among the clinical risk factors, only family history was significantly associated with BMD, showing a negative correlation across all Z-scores. This supports the hypothesis that genetic predisposition influences both hip joint development and skeletal mineralization [50]. The stronger association with lumbar spine Z-scores suggests a greater familial impact on trabecular-rich bone, which is more metabolically active and sensitive to genetic influences during growth [35].

Contrary to expectations, objectively measured physical activity levels did not correlate with BMD in this cohort. This differs from evidence in healthy children, where moderate-to-vigorous activity is associated with greater bone accrual [51]. Possible explanations include insufficient activity intensity, altered biomechanics from residual hip abnormalities, gait asymmetries, or functional adaptations that limit the osteogenic stimulus despite activity [40,52]. Also, this could partially reflect the constraints of accelerometry in children with DDH, as altered gait mechanics, asymmetry, and reduced engagement in vigorous activity could affect the device’s accuracy in capturing the true osteogenic stimulus generated by physical activity.

Muscle strength analysis revealed that total strength of the affected or non-affected limb alone was not significantly associated with BMD. Instead, the strength symmetry index, reflecting the ratio of affected-to-nonaffected limb strength, showed strong positive correlations with subtotal, total, and lumbar Z-scores, and emerged as the sole independent predictor in multivariate models. This underscores the importance of balanced bilateral muscle function for optimal skeletal loading, consistent with the mechanostat theory [39]. Asymmetrical loading, common in DDH due to joint instability, surgical history, or pain avoidance, likely reduces mechanical strain on the affected side, limiting osteogenesis and contributing to lower bone density [38].

While traditional predictors such as age, BMI, calcium intake, and family history showed univariate associations with BMD, their influence diminished in multivariate analyses, suggesting that functional asymmetry may override these factors in this clinical population. The high explanatory power of the models (R^2^ = 0.737–0.950) indicates that the included variables account for most of the variability in BMD, but the dominant role of muscle strength symmetry suggests that rehabilitation programs targeting bilateral muscle balance and symmetrical weight-bearing could be the most effective strategies for preserving bone health in children with DDH. Evidence from other pediatric populations supports the benefit of such targeted interventions in improving skeletal loading patterns and preventing bone loss [33].

### Study Limitations

The current study has a number of limitations. The sample size was relatively small, which restricts the ability to generalize the findings. There was no age-matched control group from our country included in the study, and the comparisons were based on normative reference data. The cross-sectional design prevents any causal inferences about the predictors of BMD. Calcium intake was evaluated through a parental survey, which could be influenced by recall bias. While children with comorbidities were excluded to minimize confounding, biochemical markers of bone metabolism, beverage consumption patterns, and pubertal hormones were not assessed. These factors may influence BMD and should be considered in future longitudinal studies. It is important to take these limitations into account when interpreting the results.

## 5. Conclusions

Children with developmental dysplasia of the hip (DDH) generally exhibit normal growth, nutritional status, and mobility, emphasizing the value of early diagnosis and proper intervention. Despite this, mild functional and structural asymmetries persist—particularly on the affected side—affecting limb strength, weight-bearing, and physical activity. Bone mineral density (BMD) remains within clinical norms overall, although slight deficits are observed in areas like the lumbar spine, likely due to uneven mechanical loading and functional imbalance. Efforts should focus on restoring symmetrical strength and function through targeted rehabilitation, promoting adequate physical activity that supports bone health, ensuring nutritional adequacy, monitoring bone density regularly in high-risk cases, and providing educational support to families. Further longitudinal research is also necessary to assess the long-term effects of DDH, and the impact of therapeutic strategies aimed at improving functional balance and skeletal outcomes.

## Figures and Tables

**Table 1:** Demographic and clinical characteristics of DDH children.

Variables	DDH Children (N= 25)
Age (years) (mean/SD)	8.48 ± 1.48
Gender: (N/%) Male Female	6 (24.0%) 19 (76.0%)
Weight (Kg) (mean/SD)	27.90 ± 9.96
Height (cm) (mean/SD)	129.23 ± 8.94
Body Mass Index (Kg/m^2^) (mean/SD)	16.29 ± 4.10
Ambulation Capacity: (N/%) Walks Independently Walks with Mobility Device	24 (96.0%) 1 (4.0%)
Birth Order: (N/%) First Born Others	6 (24.0%) 19 (76.0%)
Type of Delivery: (N/%) Cesarean Section Normal Delivery	11 (44.0%) 14 (56.0%)
Presentation: (N/%) Breech Presentation Normal Presentation	4 (16.0%) 21 (84.0%)
Laterality: (N/%) Unilateral Bilateral	24 (96.0%) 1 (4.0%)
Affected side: (N/%) Right Left Both	15 (60.0%) 9 (36.0%) 1 (4.0%)
Family History: (N/%) Positive Negative	13 (52.0%) 12 (48.0%)
Foot Deformities: (N/%) Present Absent	3 (12.0%) 22 (88.0%)
Associated Problems: (N/%) Present Absent	4 (16.0%) 21 (84.0%)
Treatment Intervention: (N/%) Surgical Conservative	18 (72.0%) 7 (28.0%)
Estimated Daily Calcium Intake (mg) (mean/SD)	1083.20 ± 285.00
Recommended Daily Calcium Intake (mg) (mean/SD)	1184.00 ± 176.61
Percentage of Estimated Daily Calcium Intake (%) (mean/SD)	93.37 ± 28.43

Abbreviations: DDH: Developmental Dysplasia of the Hip.

**Table 2:** Anthropometric measurements, physical activity, and isometric muscle strength in DDH children.

	Variables (mean/SD)	DDH Children (N = 25)
Anthropometric measurements	Long Measurements Discrepancy Femur (cm)	0.36 ± 0.47
	Long Measurement Discrepancy Tibia (cm)	0.34 ± 0.47
	Round Measurements Discrepancy Thigh (cm)	0.36 ± 0.45
	Round Measurement Discrepancy Leg (cm)	0.24 ± 0.41
	Weight Bearing on Affected (kg)	13.84 ± 5.31
	Weight Bearing on Nonaffected (kg)	14.26 ± 4.86
	Interside Difference (kg)	−0.42 ± 2.10
	Symmetry Index (%)	0.97 ± 0.15
Physical activity	Total Steps (number)	35869.88 ± 18235.56
	Step Rate (step/min)	8.75 ± 3.95
	Total Sedentary Time (%)	52.48 ± 10.56
	Total Time in Light Activities (%)	42.67 ± 8.80
	Total Time in Moderate Activities (%)	4.61 ± 3.22
	Total Time in Vigorous Activities (%)	0.14 ± 0.19
Isometric muscle strength	Nonaffected Hip Flexors (pounds)	18.70 ± 3.37
	Affected Hip Flexors (pounds)	15.45 ± 3.23
	Nonaffected Hip Extensors (pounds)	20.30 ± 4.35
	Affected Hip Extensors (pounds)	17.71 ± 3.80
	Nonaffected Hip Abductors (pounds)	17.64 ± 3.31
	Affected Hip Abductors (pounds)	15.60 ± 2.89
	Nonaffected Hip Adductors (pounds)	19.63 ± 4.38
	Affected Hip Adductors (pounds)	16.75 ± 3.25
	Nonaffected Knee Flexors (pounds)	15.40 ± 2.82
	Affected Knee Flexors (pounds)	13.91 ± 2.88
	Nonaffected Knee Extensors (pounds)	20.75 ± 3.34
	Affected Knee Extensors (pounds)	18.52 ± 3.16
	Nonaffected Strength Index (pounds)	112.43 ± 16.74
	Affected Strength Index (pounds)	97.96 ± 15.58
	Strength Symmetry Index (%)	0.87 ± 0.04

**Table 3:** Dual-energy X-ray absorptiometry (DXA) measurements in DDH children.

Variables	DDH Children (N = 25)
Subtotal BMC (g)	611.59 ± 157.06
Subtotal BMD (g/cm^2^)	0.61 ± 0.09
Head BMC (g)	473.26 ± 123.24
Head BMD (g/cm^2^)	1.76 ± 0.18
Total BMC (g)	993.65 ± 258.79
Total BMD (g/cm^2^)	0.83 ± 0.08
Subtotal Z-Score	−1.18 ± 1.58
Total Z-Score	0.99 ± 1.16
Total Lumbar (L1-L4) BMC (g)	16.57 ± 5.04
Total Lumbar (L1-L4) BMD (g/cm^2^)	0.50 ± 0.11
Total Lumbar (L1-L4) Z-Score	−1.41 ± 1.72

Abbreviations: BMC: Bone Mineral Content; BMD: Bone Mineral Density.

**Table 4:** Correlations between Dual-energy X-Ray absorptiometry (DXA) Z-scores and DDH children’s characteristics and outcome measures.

Pearson Correlation/Spearman Correlation					
Children Characteristics and Outcome Measures			Subtotal Z-Score	Total Z-Score	Total Lumbar Z-Score
Clinical Characteristics	^a^ Age	r	0.501	0.465	0.435
		P	0.011 *	0.019 *	0.030 *
	Weight	r	0.516 **	0.251	0.469 *
		P	0.008	0.227	0.018
	Height	r	0.481 *	0.192	0.414 *
		P	0.015	0.358	0.040
	Body Mass Index	r	0.443*	0.194	0.398 *
		P	0.027	0.352	0.049
	Family history	r	0.567 **	0.551 **	0.784 **
		P	0.002	0.004	0.0001
	Estimated Daily Calcium Intake	r	0.494 *	0.643 **	0.420 *
		P	0.012	0.001	0.037
	Percentage of Estimated Daily Calcium Intake	r	0.390	0.542 **	0.321
		P	0.054	0.005	0.118
Anthropometric Measurements	Long Measurements Discrepancy Femur	r	0.153	0.209	0.217
		P	0.466	0.317	0.298
	Long Measurement Discrepancy Tibia	r	−0.166	−0.123	−0.098
		P	0.428	0.558	0.642
	Round Measurements Discrepancy Thigh	r	0.260	0.222	0.330
		P	0.210	0.287	0.107
	Round Measurement Discrepancy Leg	r	−0.121	−0.070	−0.029
		P	0.564	0.738	0.889
	Weight Bearing on Affected	r	0.458 *	0.250	0.434 *
		P	0.021	0.228	0.030
	Weight Bearing on Nonaffected	r	0.439 *	0.189	0.371
		P	0.028	0.366	0.068
	Weight Bearing Interside Difference	r	0.201	0.181	0.137
		P	0.335	0.387	0.513
	Weight Bearing Symmetry Index	r	0.208	0.203	0.192
		P	0.320	0.331	0.357
Physical Activity	^a^ Step Rate	r	−0.199	−0.082	−0.127
		P	0.341	0.698	0.547
	Percentage of Total Sedentary Time	r	0.001	−0.106	−0.105
		P	0.996	0.613	0.617
	Percentage of Total Time in Light Activities	r	−0.046	0.104	0.101
		P	0.826	0.622	0.632
	^a^ Percentage of Total Time in Moderate Activities	r	−0.110	−0.009	−0.092
		P	0.601	0.967	0.662
	^a^ Percentage of Total Time in Vigorous Activities	r	0.160	0.132	0.121
		P	0.445	0.529	0.565
Isometric Muscle Strength	Nonaffected Hip Flexors	r	0.055	−0.069	0.062
		P	0.795	0.744	0.769
	Affected Hip Flexors	r	0.156	0.109	0.140
		P	0.457	0.604	0.503
	Nonaffected Hip Extensors	r	−0.066	0.006	0.033
		P	0.753	0.977	0.875
	Affected Hip Extensors	r	0.204	0.206	0.287
		P	0.328	0.323	0.164
	Nonaffected Hip Abductors	r	0.158	0.049	0.188
		P	0.449	0.816	0.368
	Affected Hip Abductors	r	0.221	0.132	0.202
		P	0.288	0.528	0.333
	Nonaffected Hip Adductors	r	−0.216	−0.172	−0.210
		P	0.299	0.411	0.314
	Affected Hip Adductors	r	0.132	0.106	0.159
		P	0.530	0.615	0.448
	Nonaffected Knee Flexors	r	0.180	0.116	0.013
		P	0.390	0.582	0.950
	Affected Knee Flexors	r	0.448 *	0.212	0.230
		P	0.025	0.308	0.269
	Nonaffected Knee Extensors	r	0.149	0.280	0.121
		P	0.477	0.176	0.564
	Affected Knee Extensors	r	0.339	0.350	0.259
		P	0.097	0.086	0.212
	Nonaffected Strength Index	r	0.029	0.028	0.030
		P	0.892	0.895	0.888
	Affected Strength Index	r	0.302	0.230	0.265
		P	0.142	0.269	0.201
	Strength Symmetry Index	r	0.967 **	0.715 **	0.818 **
		P	0.000	0.004	0.003

Abbreviations: r: Correlation coefficient; P: Significant correlation; ^a^: Spearman Correlation. *: Correlation is significant at the 0.05 level; **: Correlation is significant at the 0.01 level.

**Table 5:** Multiple Linear Regression Model for the prediction of the Dual-energy X-Ray absorptiometry (DXA) Z-scores in DDH children.

	Predictors	Standardized Coefficients Beta	Sig.	Model Summary
Subtotal Z-score	Age	0.117	0.125	R Square (0.958) Sig. (<0.001 *)
	Body Mass Index	0.121	0.536	
	Weight Bearing on Affected	0.047	0.771	
	Weight Bearing on Nonaffected	−0.207	0.256	
	Estimated Daily Calcium Intake	0.001	0.989	
	Family History	−0.058	0.484	
	Affected Knee Flexors	−0.090	0.244	
	Strength Symmetry Index	1.000	<0.001 *	
Total Z-score	Age	−0.059	0.713	R Square (0.737) Sig. (<0.001 *)
	Estimated Daily Calcium Intake	0.293	0.213	
	Percentage of Estimated Daily Calcium Intake	0.254	0.272	
	Family History	0.160	0.308	
	Strength Symmetry Index	0.425	0.023 *	
Total Lumbar Z-score	Age	0.126	0.425	R Square (0.762) Sig. (<0.001 *)
	Body Mass Index	0.019	0.954	
	Weight Bearing on Affected	−0.002	0.995	
	Estimated Daily Calcium Intake	0.156	0.266	
	Family History	0.280	0.091	
	Strength Symmetry Index	0.499	0.014*	

*: Significant at 0.05.

## Data Availability

The data supporting the findings and analyzed during this study are available from the corresponding author upon reasonable request.

## Abbreviations

The following abbreviations are used in this manuscript:

DDH	Developmental Dysplasia of the Hip
DXA	Dual-Energy X-Ray Absorptiometry
BMD	Bone Mineral Density
BMC	Bone Mineral Content
KFHU	King Fahd Hospital of the University
BMI	Body Mass Index
BMI	Physical Activity
IMS	Isometric Muscle Strength
HHD	Handheld Dynamometer
IOF	International Osteoporosis Foundation
MVPA	Moderate-To-Vigorous Physical Activity
SD	Standard Deviation
